# TAM and MUSIC Approach for Impact-Source Localization under Deformation Conditions

**DOI:** 10.3390/s20113151

**Published:** 2020-06-02

**Authors:** Zhenghao Zhang, Yongteng Zhong, Jiawei Xiang

**Affiliations:** College of Mechanical and Electrical Engineering, Wenzhou University, Wenzhou 325035, China; zhangzhenghao@stu.wzu.edu.cn (Z.Z.); jwxiang@wzu.edu.cn (J.X.)

**Keywords:** Toeplitz approximation method, multiple-signal classification, composite structures, deformation conditions, impact-source localization, Lamb waves

## Abstract

As an impact-source-localization technique, Lamb waves are commonly used to detect low-velocity impact in composite structures. However, the performance of Lamb waves is susceptible under deformation conditions. In this paper, a novel approach combined the Toeplitz approximation method (TAM) and multiple-signal classification (MUSIC) (TAM-MUSIC) to improve impact-source-localization (angle and distance in polar coordinates) accuracy under deformation conditions. The method divided a two-dimensional search of direction and distance into two one-dimensional searches. The impact direction was calculated by the TAM, which was introduced into the steering vector of MUSIC to estimate the distance by scanning the whole monitoring area. An epoxy laminate plate experiment showed that the phase and amplitude of uniform linear-array signals had different average plate curvature that led to poor impact-source-localization accuracy using the MUSIC method. TAM provided high-resolution direction-finding capability, suitable for the pretreatment of Lamb waves. Results showed that the present method, with a small amount of computation and low memory requirement, had higher location-estimation accuracy than that of traditional methods under deformation conditions.

## 1. Introduction

Compared with common metallic materials, composite materials have properties that increase their applicability in engineering structures [1]. However, their structural mechanical properties decrease as a result of low-velocity impact in actual environments [2,3,4]. Thus, it is important to locate impact sources for structural-health monitoring (SHM) [5,6,7]. Lamb waves are considered prominent and promising tools for composite structures in the SHM field [8,9].

Many localization methods based on Lamb waves using the sparse-sensor array have been proposed for SHM, including a triangulation technique [10], four-point arch localization [11], and a time-reversal imaging method [12]. However, these methods are readily influenced by the orthotropic performance of the composite during application. On the other hand, several methods were developed for impact-source estimation using compact-sensor arrangement, such as the spatial filter-based method [13] and the phased-array-based method [14]. Schmidt et al. [15] described the multiple-signal-classification (MUSIC) method, based on the eigenvalue decomposition of the sample-covariance matrix, as a breakthrough space-spectrum algorithm to calculate the direction of arrival (DOA). Yan et al. [16] combined the MUSIC algorithm with wavelet-packet analysis to identify directions of leak sources in large-pressure vessels. Yang et al. [17] used the MUSIC method and time delay of arrival to calculate direction and distance to locate the impact source in a plate. He et al. [18] developed an imaging algorithm using time-reversal operator decomposition and the MUSIC algorithm to image damage in a metallic plate. Yuan et al. [19,20] proposed a two-dimensional near-field MUSIC algorithm to estimate the angle and distance in polar coordinates of the impact source. According to a propagation model of guided waves, Zuo et al. [21] developed a model-based 2D MUSIC algorithm for damage identification on the basis of near-field assumption. Bao et al. [22] proposed an improved MUSIC method based on transmitter beamforming and weighted-image fusion for the corrosion monitoring of aluminum plates.

Although the performance advantages (superior resolution and high accuracy) of MUSIC are substantial, they are achieved at considerable cost in computation (searching over space) and storage (sensor-array data). To deal with this problem, Roy et al. [23,24] presented an estimation of signal parameters via rotational-invariance techniques (ESPRIT) that have computational and numerical advantages to obtain the DOA. EI Kassis et al. [25] used expectation maximization and the ESPRIT method to solve the problem of DOA estimation with nonuniform linear arrays. Kung et al. [26] proposed a Toepitz approximation method for retrieving sinusoidal processes from noisy measurements. Because of its low sensitivity to data perturbation, the Toeplitz approximation method (TAM) algorithm was also used for source-direction finding in a noisy environment [27]. Rao et al. [28] developed a state-space approach on the basis of a model for certain nonlinear-estimation problems, and applied it in direction finding and damped-sinusoid retrieval.

The structural deformation of composite materials commonly occurs as a result of processing, assembly, vibration, impact, and high temperatures in a real environment. In aerospace, external airflow changes cause wing deformation and lead to signal perturbation, bringing new challenges to impact-source localization. Some researchers introduced artificial neural networks into impact detection (location and force reconstruction). Lee et al. [29] carried out a biaxial test of a composite tube under combined torsion, and used artificial neural networks to predict failure strength. Labossiere et al. [30] used neural networks to obtain the example failure envelope of a typical fiber-reinforced material for predicting the failure of anisotropic materials. Al-Assaf et al. [31] used artificial neural networks to predict the fatigue behavior of unidirectional glass fiber/epoxy composite laminae under tension–tension and tension–compression loading. Two different back-propagation neural networks were developed to represent the nonlinear stress–strain behavior of graphite–epoxy laminates under monotonic and cyclic loadings by Pidaparti et al. [32]. A back-propagation neural network was used to predict the fatigue strength of composite materials by Aymerich et al. [33]. However, the application of artificial neural networks needs large amounts of data, and the training process needs huge computing and storage resources.

Combining the advantage of low sensitivity to signal changes and low computation, this paper presents a TAM-MUSIC approach for composite structures under deformation conditions. In the present method, TAM is used to estimate the direction of the impact source, and distance is calculated by a one-dimensional search in the spatial spectrum of MUSIC. This paper is structured as follows: experiment and sensor-array-signal analyses are given in Section 2. In Section 3, a TAM-MUSIC approach is proposed for locating the impact source of the composite structures under deformation conditions. Experiment investigations of impact-source-localization methods under deformation conditions are performed in Section 4. Section 5 gives the conclusion.

## 2. Experiment Setup and Signal Analysis

### 2.1. Experiment Setup of Epoxy Laminate Plate under Deformation Conditions

**Figure 1:**
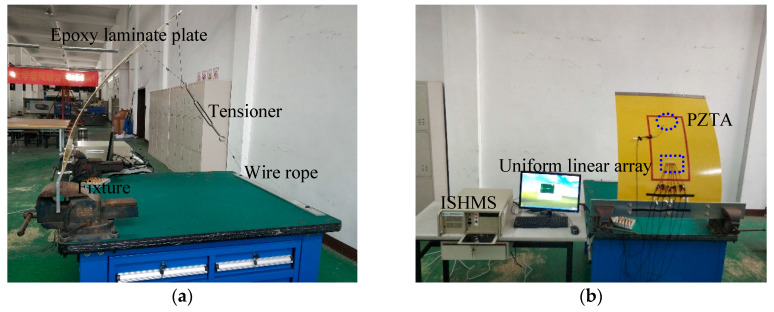
Deformation experiment. (**a**) Bent epoxy laminate plate; (**b**) experiment setup.

**Figure 2:**
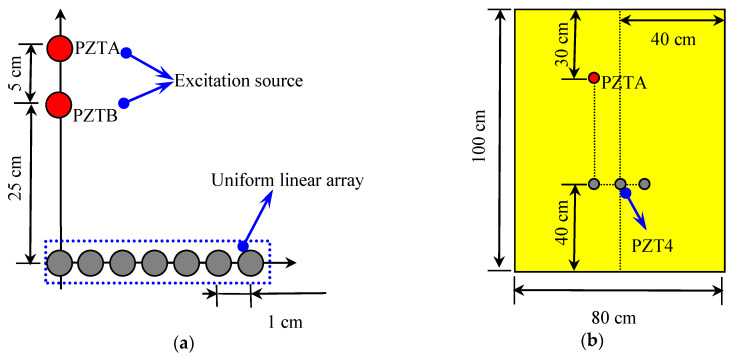
Deformation experiment: (**a**) sensor arrangement; (**b**) plate sensors.

The setup of the deformation experiment is shown in Figure 1, including a deformation system, an integrated structural-health-monitoring scanning system (ISHMS), and an epoxy laminate plate. The deformation system included a workbench, fixture, aluminum plate, aluminum bar, wire rope, and tensioner. The dimensions of the aluminum plates were 120 × 12 × 0.5 cm. One side of the epoxy laminate plate was fixed with two aluminum plates using screws, and another side of the plate was pulled by the wire rope and tensioner. The total range of the tensioner was 16.5 cm, and the dimensions of the aluminum bar were 100 × 8 × 0.5 cm. The dimensions of the epoxy laminate plate were 100 × 80 × 0.2 cm, and the ply sequence was [0_2_/90_4_/0_2_]. The thickness of each ply was 0.125 mm. The uniform linear array (ULA) consisted of seven lead zirconate titanate (PZT) piezoelectric ceramic sensors that were labeled as PZT1, PZT, …, PZT7, respectively, from left to the right. The space between adjacent PZTs was 1 cm. The diameter and thickness of the PZT sensors were 8 and 0.48 mm, respectively. Two more excitation sources, labeled PZTA and PZTB, were used to simulate impact signals. The distance between PZT1 and PZTB was 25 cm, and the space between PZTA and PZTB was 5 cm as shown in Figure 2a. PZT4 was located on the center line of the plate, as shown in Figure 2b. Sampling rate was set at 10 MHz, and excitation frequency at 50 kHz. Sampling length was 5000, including 500 pretrigger samples.

### 2.2. Sensor-Array Signals Influenced by Deformation Conditions

**Figure 3:**
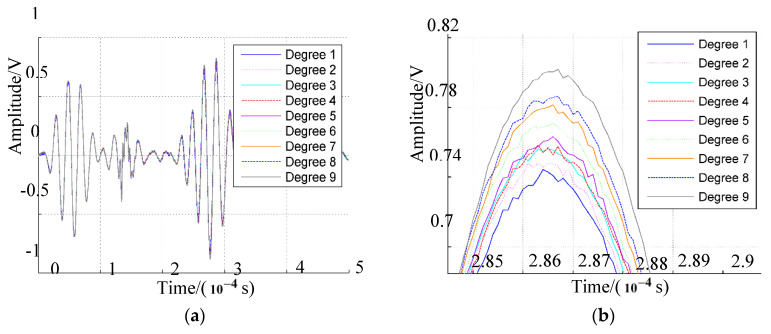
Lead zirconate titanate 4 (PZT4) signals in deformation experiment: (**a**) PZT4 signal when PZTA was excited; (**b**) highest wave peak.

**Figure 4:**
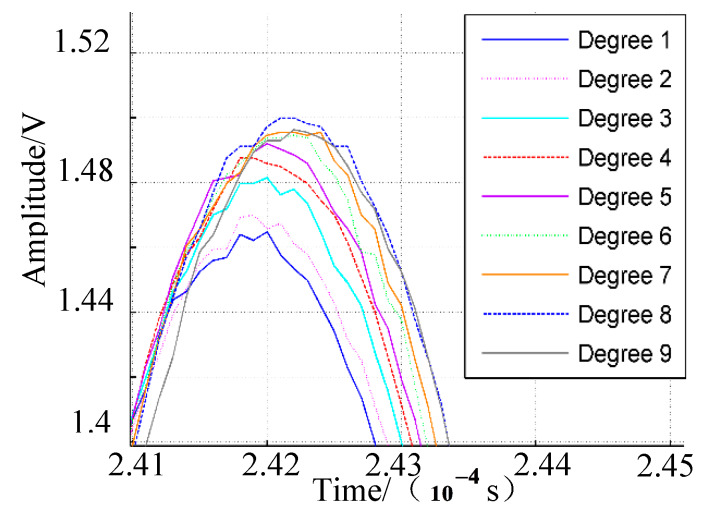
Highest wave peak when PZTB was excitation sensor.

Sensor-array signals under different deformation conditions were compared to investigate the deformation effect. Deformation of the epoxy laminate plate was controlled by a tensioner incrementally stretched inward by 2 cm to change the average curvature of the plate. The deformation degree of the plate was set at degree 1 when the tensioner was totally opened, as shown in Figure 1a. The deformation states of the plate were labeled Degree 1–9 depending on curvature over time. The average curvatures of the plate corresponding to deformation degrees from 1 to 9 were 2.41, 2.31, 2.21, 2.16, 2.06, 2.02, 1.96, 1.91, and 1.83 rad/m, respectively. The average curvature decreased when the plate tended towards greater curvature. The response signals of PZT4 under deformation degrees 1 to 9 when the PZTA was excited, are shown in Figure 3a. In order to intuitively determine the deformation effect on response signals, the variation of the highest wave peaks in direct waves was observed, as shown in Figure 3b. The highest wave peak increased with deformation degree when PZTB was excited as shown in Figure 4. The PZT4 signal had the lowest amplitude under Degree 1 deformation, and the highest amplitude in deformation under Degree 9. As the deformation degree increased from 1 to 9, the signal amplitude increased accordingly, so that the extent of direct waves increased with the increased deformation degree of the plate. Changes in response signals are related to errors associated with impact-source localization based on the increase in array-signal processing.

Guided waves also exist in thin-walled structures such as rods, tubes, and shells. The deformation plate could be considered as a part of a hollow circular cylinder. There are three modes of guided-wave propagation in a hollow circular tube: L mode with only axial and radial displacement components, T mode with only circumferential displacement components, and F mode with displacements in three directions and coupled [34,35]. Boundary conditions used in solving wave modes in infinite round tubes are dependent on their inner and outer diameters. Changing diameters induced by deformation lead to confusion of the boundary conditions. A change in the mode of guided waves is caused by deformation. Thus, the received sensor-array signals were different compared to previous signals, and the MUSIC algorithm failed to locate the impact.

## 3. TAM-MUSIC Approach

**Figure 5:**
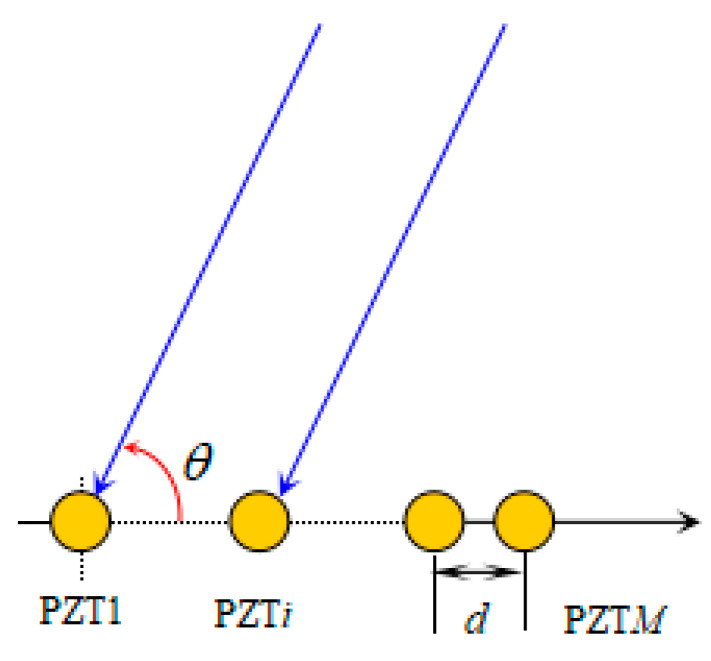
Toeplitz approximation method (TAM) diagram.

The deformation experiment investigated if the sensor-array signals had a finite change, which brought new challenges concerning the health monitoring of composite structures. To deal with this problem, TAM-MUSIC is proposed to estimate the impact source. A ULA consisted of *M* PZTs with interelement spacing d, as shown in Figure 5. Supposing a far-field impact-signal direction with respect to the ULA is θ.

PZT1 was set to be the reference sensor, and the signal can be expressed as

(1) $$s_{1}(t)=v(t)\exp[j(\omega_{0}t+\phi (t))],$$

where v(t) is the amplitude of the impact signal, $\omega_{0}$ is the center frequency of the impact signal, ϕ(t) is the phase of the impact signal.

The response signal of PZTi (1≤i≤M) was calculated as:

(2) $$s_{i}(t)=s_{1}(t)\exp(j\omega_{0}\tau_{i})+n_{i}(t)=z_{i}(t)+n_{i}(t),$$

where $\tau_{i}$ is the impact-signal-arriving time difference between PZTi and PZT1, $n_{i}(t)$ is the background noise.

Thus, $z_{i+1}(t)=Dz_{i}(t)$, where rotation invariant factor D=exp(jβ), β=2πdcosθ/λ. The signals output from the whole ULA can be presented as:

(3) $$Z(t)={\left[z_{1}(t),z_{2}(t),\cdots ,z_{M}(t)\right]}^{T}$$

Then, the covariance matrix without the noise of the whole sensor array is defined as

(4) $$\begin{array}{l} R_{0}=[Z(t)Z^{H}(t)]\\ \;=\left[\begin{array}{c} 1\\ D\\ \vdots \\ D^{M-1} \end{array}\right]R_{Z}\left[\begin{array}{cccc} 1 & D^{-1} & \cdots & D^{-M+1} \end{array}\right]\\ \;=BR_{Z}B^{H} \end{array}$$

where $R_{Z}$ is the covariance matrix of the PZT1 signal.

The actual covariance matrix of ULA is decomposed by singular value decomposition (SVD):

(5) $$\begin{array}{ll} {\hat{R}}_{1} & ={\hat{R}}_{0}+{\hat{R}}_{N}\\ & =\left[\begin{array}{cc} {\hat{U}}_{S} & {\hat{U}}_{N} \end{array}\right]\left[\begin{array}{cc} {\hat{\Sigma }}_{S} & 0\\ 0 & {\hat{\Sigma }}_{N} \end{array}\right]\left[\begin{array}{c} {\hat{V}}_{S}^{H}\\ {\hat{V}}_{N}^{H} \end{array}\right] \end{array}$$

In the ideal case, the covariance matrix is a Toeplitz matrix. However, the signal is easily also influenced by other factors. Thus, matrix ${\hat{R}}_{2}$ is structured to replace $R_{0}$ in Equation (5):

(6) $${\hat{R}}_{2}={\hat{U}}_{S}{\hat{\Sigma }}_{S}{\hat{V}}_{S}^{H}$$

Matrix-C-based rotational invariance subspace is defined as

(7) $$C=U_{S}\Sigma_{S}^{1/2}.$$

The first M−1 rows and last M−1 rows are $C_{1}={\left[\begin{array}{cccc} 1 & D & \cdots & D^{M-2} \end{array}\right]}^{T}$ and $C_{2}={\left[\begin{array}{cccc} D & D^{2} & \cdots & D^{M-1} \end{array}\right]}^{T}$. The relationship between $C_{1}$ and $C_{2}$ is:

(8) $$C_{1}D=C_{2}\Rightarrow U_{S1}\Sigma_{S}^{1/2}D=U_{S2}\Sigma_{S}^{1/2}$$

where $U_{S1}$ is the first M−1 rows of $U_{S}$, $U_{S2}$ is the last M−1 rows of $U_{S}$.

Direction angle θ of the impact source can be calculated using D from Equation (8) as

(9) $$\theta =\arccos(\frac{\lambda \ln D}{2j\pi d}).$$

The proposed 2D-MUSIC algorithm in [19] is a two-dimensional (angle and distance) search method to locate impact sources. With DOA estimation, the distance of the impact source can be calculated and used in a one-dimensional (distance) search. The response signal of PZTi in ULA can be denoted as

(10) $$x_{i}(t)=\frac{r}{r_{i}}x_{1}(t)\exp(j\omega_{0}\tau_{i})+n_{i}(t),\;i=1,2,\cdots ,M$$

where $n_{i}(t)$ is the background noise, and r is the distance between impact source and response sensor PZT1. The distance between impact source and PZTi is calculated as

(11) $$r_{i}=\sqrt{r^{2}+d^{2}{\left(i-1\right)}^{2}-2rd(i-1)\cos\theta }.$$

With angle estimation, the steering vector is denoted as

(12) $$a_{i}(r)=\frac{r}{r_{i}}\exp(j\omega_{0}\tau_{i}),$$

where the $\tau_{i}$ is defined as arriving time difference between PZTi and PZT1, $\tau_{i}=(r-r_{i})/c$. For the whole ULA, signals can be presented as

(13) $$X(t)=A(r)x_{1}(t)+N(t),$$

where

(14) $$\begin{array}{l} X(t)={\left[x_{1}(t),x_{2}(t),\cdots ,x_{M}(t)\right]}^{T}\\ A(r,\theta )={\left[a_{1}(r),a_{2}(r),\cdots ,a_{M}(r)\right]}^{T}\\ N(t)={\left[n_{1}(t),n_{2}(t),\cdots ,n_{M}(t)\right]}^{T} \end{array}$$

The covariance matrix of the response signal vector is

(15) $$\hat{R}=\frac{1}{N}XX^{H}=U_{S}\Sigma_{S}U_{S}^{H}+U_{N}\Sigma_{N}U_{N}^{H},$$

where superscript H denotes the conjugate transpose, $U_{S}$ denotes the signal subspace spanned by the eigenvector matrix corresponding to the largest eigenvalue, $U_{N}$ denotes the noise subspace spanned by the eigenvector matrix corresponding to those small eigenvalues, and N is the sampling length.

The spatial spectrum is expressed as

(16) $$P_{}(r)=\frac{1}{A^{H}(r)U_{N}U_{N}{}^{H}A(r)}.$$

**Figure 6:**
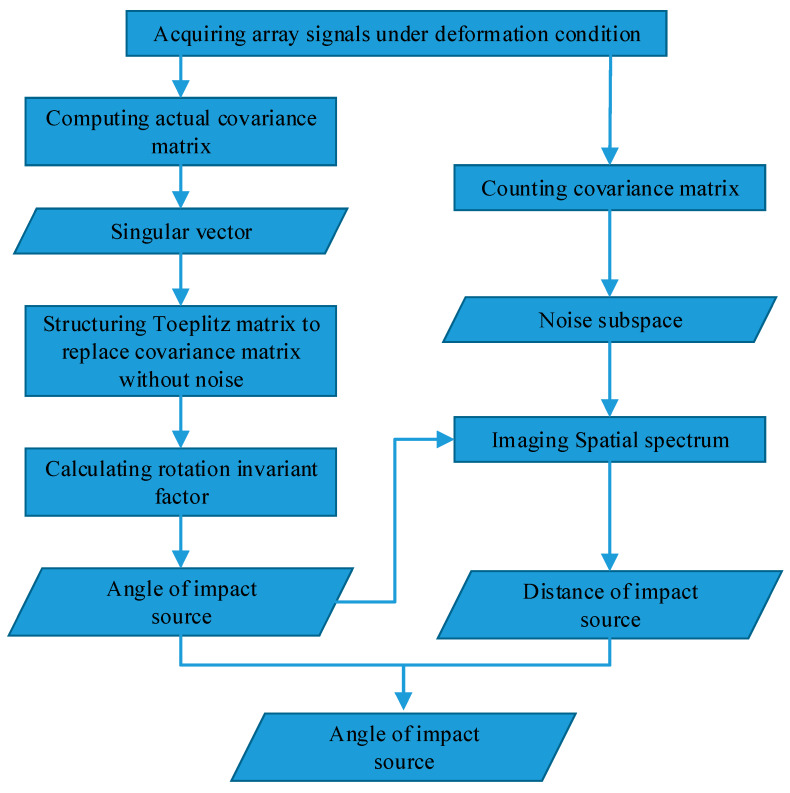
Block diagram of present TAM-MUSIC approach.

The locating-impact-source process of the proposed TAM-MUSIC approach is shown in Figure 6.

## 4. Impact-Source-Localization Results under Deformation Conditions

**Figure 7:**
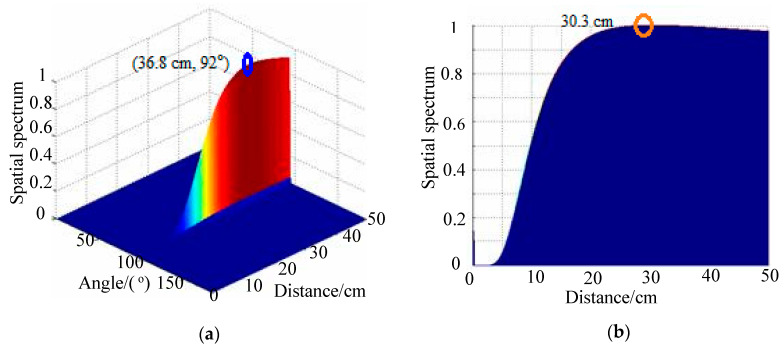
Spatial spectra of two methods under deformation degree 5 when PZTA was excited: (**a**) 2D-MUSIC algorithm; (**b**) TAM-MUSIC method.

**Figure 8:**
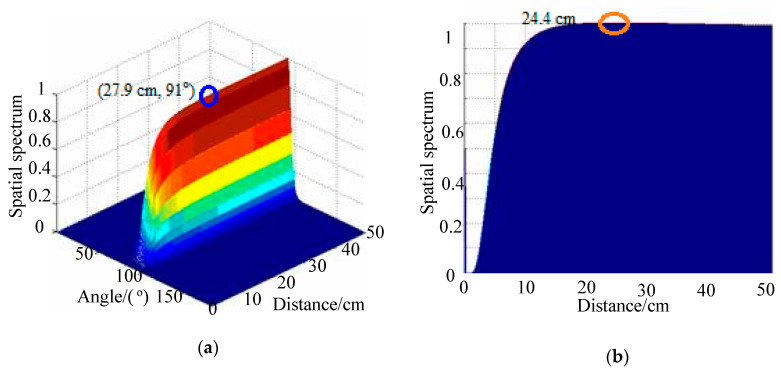
Spatial spectra of two methods under deformation degree 5 when PZTB was excited: (**a**) 2D-MUSIC algorithm; (**b**) TAM-MUSIC method.

In this section, 2D-MUSIC algorithm results are compared with results using the TAM-MUSIC approach for localization under deformation conditions. The positions of excitation sources PZTA and PZTB in polar coordinates were (30.0 cm, 90°) and (25.0 cm, 90°), respectively. Figure 7a shows the spatial spectrum of the 2D-MUSIC algorithm under deformation degree 5 when the PZTA was excited, where the distance error was 6.8 cm and the angle error was 2°. The angle of impact direction was directly calculated used TAM. The angle introduced the steer vector of the 2D-MUSIC, thereby changing the two-dimensional search into a one-dimensional search. Lastly, the distance of the impact position was estimated by a one-dimensional search using the TAM-MUSIC approach. With the impact angle, the impact distance was searched in a monitoring area from 0 to 50 cm with a step of 0.1 cm. Using the present approach, the angle result was 91°, and the distance research result was 30.3 cm, as shown in Figure 7b. The relative distance error and the length between PZTA and PZT1 were reduced from 22.7% to 1%. The localization result of the 2D-MUSIC algorithm under the deformation of Degree 5 when the PZTB source was excited is shown in Figure 8a. The distance error was 2.9 cm, and the angle error 1°. Using the TAM-MUSIC approach, the angle result was 91°, and the distance result was 24.4 cm, as shown in Figure 8b. The relative error was reduced from 11.6% to 2.4%. The localization results of the 2D-MUSIC algorithm and the TAM-MUSIC approach under deformation conditions are listed in Table 1, where the computing times are also shown. Using the 2D-MUSIC algorithm, the maximal errors of distance and angle estimation were 14.6 cm and 2°, respectively. Distance error was less than 2 cm, and angle error was less than 1° in ther localization results using the TAM-MUSIC approach. The computing times of the TAM-MUSIC approach were almost one-quarter of those of the 2D-MUSIC algorithm.

**Figure 9:**
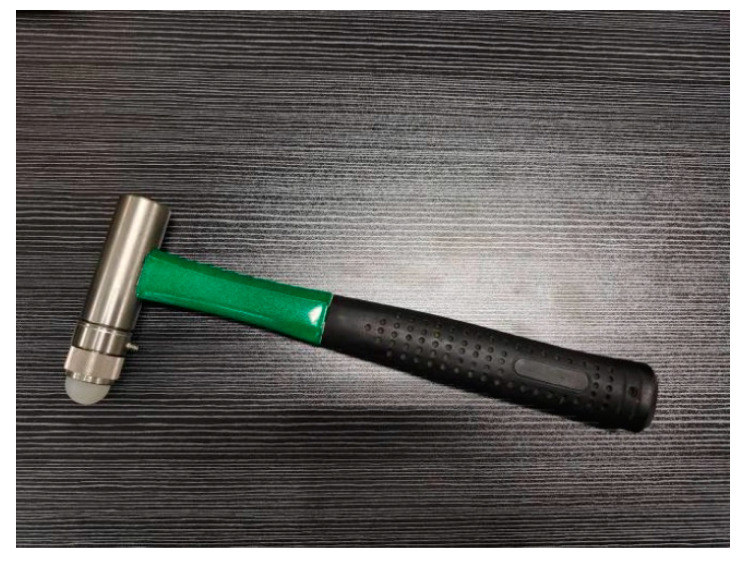
Force hammer.

The impact signal simulated by the excitation source was a narrow band signal in our experiments, but the impact signal in the actual environment is also commonly a wide band signal. In order to verify the performance of the present approach in a real environment, the force hammer in Figure 9 was used to simulate real impact when the plate was under degree 3 of deformation.

**Figure 10:**
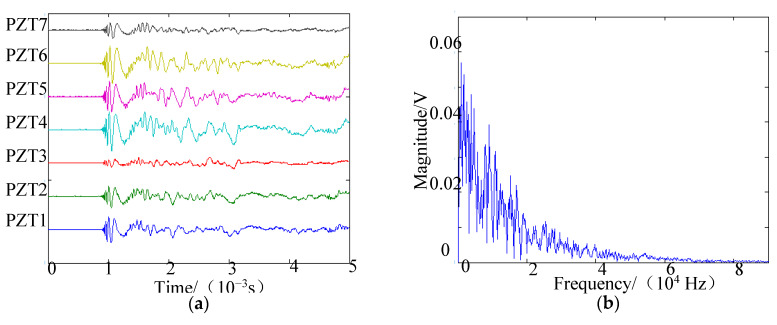
Sensor signals when impact occurred: (**a**) array-sensor signals; (**b**) frequency spectrum of impact signal obtained by PZT1.

**Figure 11:**
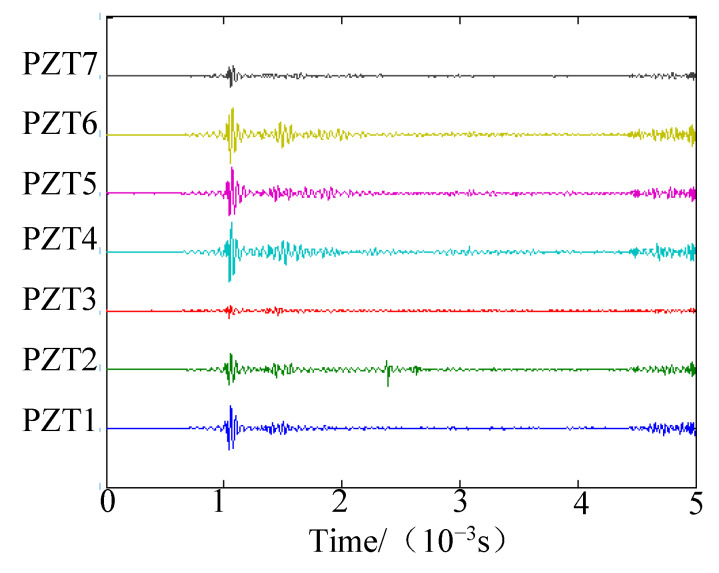
Narrow-band-impact signals.

**Figure 12:**
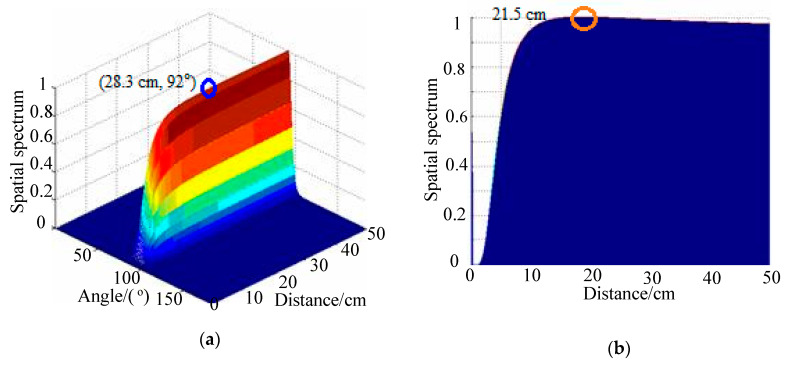
Spatial spectra of two methods when real impact occurs: (**a**) 2D-MUSIC algorithm; (**b**) TAM-MUSIC method.

The position of the impact source simulated by the force hammer was (20.0 cm, 90°). PZT1 was set as the trigger channel, and the trigger voltage was 0.5 V in the experiments. Sampling rate was set at 2 MHz, and the sampling length at 10,000 including 2000 pretrigger samples. Sensor signals are shown in Figure 10a, and the frequency spectrum of the PZT1 signal is given in Figure 10b. The energy of the impact signal was mainly in the frequency band from 0 to 70,000 Hz. The narrow band signal could be decomposed from the original signal if wavelet transform were used [36,37]. According to [19], Gabor wavelet transform was used to decompose the impact signals, and 45 kHz was selected as the central frequency of the narrow band signal. The narrow-band-impact signals are shown in Figure 11. Figure 12a shows the spatial spectrum of the 2D-MUSIC algorithm under deformation degree 3, where distance error was 8.3 cm and angle error was 2°. Using the present approach, the angle result was 91°, and the distance research result was 21.5 cm, as shown in Figure 12b.

## 5. Conclusions and Future Works

The deformation effects of composite structures (epoxy laminate plates) on Lamb waves were investigated. Phase and amplitude of uniform linear array signals changed when the plate was bent with different average curvatures. Therefore, the impact-source-localization accuracy of the traditional MUSIC method was enormously reduced. To solve this problem, a TAM-MUSIC algorithm for impact-source localization was proposed. Compared with the 2D-MUSIC algorithm, the impact-source-localization (angle and distance in polar coordinates) results using the present method had higher accuracy when the plate status changed. The distance and direction errors of the TAM-MUSIC algorithm were estimated at less than 2 cm and 2°, respectively. Moreover, the average relative error of the prediction distance error, and the length between excitation source and PZT1 were reduced from 15.6% to 7.15%. Furthermore, because the Lamb waves were divided, a two-dimensional search (angle and distance in polar coordinates) was converted into two one-dimensional searches (angle or distance in polar coordinates), so that only small amounts of computation and low computer memory were required in the present method. Average computing time was reduced by 0.9 s using the present approach, providing the possibility for real-time monitoring under deformation conditions. Therefore, we conclude that the present method has certain essential advantages, such as the higher accuracy of impact-source localization under deformation conditions, reduced computation, and low memory requirements. Further research is required to explain the physical mechanisms concerning structural-deformation effects on Lamb wave signals and impact localization to improve impact-localization accuracy.

## Figures and Tables

**Table 1:** Localization results of two methods under deformation. MUSIC, multiple-signal classification.

		2D-MUSIC Results		2D-MUSIC Errors		2D-MUSIC Computing Time	TAM-MUSIC Results		TAM-MUSIC Errors		TAM-MUSIC Computing Time
Deformation		${\hat{r}}_{1}/\mathrm{cm}$	${\hat{\theta }}_{1}/{}^{\circ}$	$E_{r1}/\mathrm{cm}$	$E_{\theta 1}/{}^{\circ}$	${\hat{T}}_{1}/s$	${\hat{r}}_{2}/\mathrm{cm}$	${\hat{\theta }}_{2}/{}^{\circ}$	$E_{r_{2}}/c$	$E_{\theta 2}/{}^{\circ}$	${\hat{T}}_{2}/s$
PZTB-1		25.9	91	0.9	1	1.2247	24.0	91	1.0	1	0.2903
PZTB-2		25.4	91	0.4	1	1.2319	24.3	91	0.7	1	0.2839
PZTB-3		25.8	91	0.8	1	1.2341	23.3	91	1.7	1	0.2867
PZTB-4		26.5	91	1.5	1	1.2316	23.3	91	1.7	1	0.2930
PZTB-5		27.9	91	2.9	1	1.2132	24.4	91	0.6	1	0.2863
PZTB-6		27.9	91	2.9	1	1.2213	25.5	91	0.5	1	0.2851
PZTB-7		29.9	91	4.9	1	1.2160	26.7	91	1.7	1	0.2962
PZTB-8		29.4	91	4.4	1	1.2085	26.5	91	1.5	1	0.3076
PZTB-9		31.3	91	6.3	1	1.2295	24.1	91	0.9	1	0.2958
PZTA-1		31.8	92	1.8	2	1.3641	30.9	91	0.9	1	0.3158
PZTA-2		34.7	92	4.7	2	1.3648	31.0	91	1.0	1	0.3336
PZTA-3		33.0	92	3.0	2	1.3632	29.6	91	0.4	1	0.3181
PZTA-4		32.5	92	2.5	2	1.3595	29.3	91	0.7	1	0.3256
PZTA-5		36.8	92	6.8	2	1.3704	30.3	91	0.3	1	0.3235
PZTA-6		36.6	92	6.6	2	1.3762	30.8	91	0.8	1	0.4555
PZTA-7		37.4	92	7.4	2	1.3664	31.4	91	1.4	1	0.3305
PZTA-8		40.7	92	10.7	2	1.3547	31.8	91	1.8	1	0.3286
PZTA-9		44.6	92	14.6	2	1.3591	31.9	91	1.9	1	0.3371
